# Isolation and Purification of Two Isoflavones from *Hericium erinaceum* Mycelium by High-Speed Counter-Current Chromatography

**DOI:** 10.3390/molecules23030560

**Published:** 2018-03-02

**Authors:** Jinzhe He, Peng Fan, Simin Feng, Ping Shao, Peilong Sun

**Affiliations:** 1 Department of Food Science and Engineering, Zhejiang University of Technology, Hangzhou 310014, Zhejiang, China; hejzgd@163.com (J.H.); iamfanpeng@163.com (P.F.); 2 Ocean College, Zhejiang University of Technology, Hangzhou 310014, Zhejiang, China; pingshao325@zjut.edu.cn

**Keywords:** *Hericium erinaceuns* mycelium, high-speed counter-current chromatography (HSCCC), genistein, daidzein

## Abstract

High-speed counter-current chromatography (HSCCC) was used to separate and purify two isoflavones for the first time from *Hericium erinaceum* (*H. erinaceum*) mycelium using a two-phase solvent system composed of chloroform-dichloromethane-methanol-water (4:2:3:2, *v*/*v*/*v*/*v*). These two isoflavones were identified as genistein (4′,5,7-trihydroxyisoflavone, C_15_H_10_O_5_) and daidzein (4′,7-dihydroxyisoflavone, C_15_H_10_O_4_), using infrared spectroscopy (IR), electro-spary ionisation mass (ESI-MS), ^1^H-nuclear magnetic resonance (NMR) and ^13^C-NMR spectra. About 23 mg genistein with 95.7% purity and 18 mg daidzein with 97.3% purity were isolated from 150 mg ethanolic extract of *H. erinaceum* mycelium. The results demonstrated that HSCCC was a feasible method to separate and purify genistein and daidzein from *H. erinaceum* mycelium.

## 1. Introduction

*Hericium erinaceum* (*H. erinaceum*), commonly called monkey head mushroom or lion’s mane mushroom, is a wood-rotting fungi that belong to the *Hericiaceae* family [1,2]. It is a traditional edible mushroom widely used as herbal medicines in East Asian countries [3]. *H. erinaceum* was reported to have many bioactivities, including anti-oxidant, immune regulatory, anti-aging, anti-microbial, anti-inflammatory, and anti-cancer activities [4,5,6,7,8]. 

Many phytochemicals, including polysaccharides, pyrones, terpenoids, phenols are present in the mycelium and fruiting bodies of *H. erinaceum* [9,10,11]. Flavonoids are found in many plant species and exhibit many bioactivities, including anti-oxidant, anti-inflammatory and anti-cancer effects [12,13]. It was reported that the methanol extract of *H. erinaceum* had strong antioxidant activities as it contained flavonoids and phenolic compounds [14]. However, specific flavonoid spices in *H. erinaceum* have still not been identified. In view of these beneficial properties, study on the separation and purification of flavonoids from *H. erinaceum* is necessary.

Many studies have been focused on the separation and purification of flavonoids from natural plants [15,16]. Most separation and purification methods are based on thin-layer chromatography and other chromatographic techniques based on a solid stationary phase, such as semi-preparative and preparative HPLC [17]. These methods were generally restricted to several disadvantages, including low-yielding, time-consuming, complex processing, high-cost, poor reproducibility, and irreversible adsorption [18,19]. High-speed counter-current chromatography (HSCCC) is an advanced technique based on liquid-liquid partitioning and has played an important role in the preparation of phytochemicals in the last decade [20]. HSCCC provides advantages by eliminating the solid support, which may cause adsorption or degradation of target compounds [18,21]. Therefore, it has been widely used for separation and purification of flavonoids, alkaloids, terpenes, polyphenols, and other natural products [22,23,24,25,26]. To the best of our knowledge, there was no report about isolating and purifying of isoflavones from *H. erinaceum* mycelium by HSCCC.

In this study, we discussed the development of the HSCCC method for the separation and purification of pure isoflavones from *H. erinaceum* mycelium. The two-phase solvent system composed of chloroform-dichloromethane-methanol-water was established. In addition, chemical structures of two purified isoflavones were further identified by infrared spectroscopy (IR), electro-spary ionisation mass (ESI-MS), ^1^H-nuclear magnetic resonance (NMR) and ^13^C-NMR spectra. As a result, two isoflavones, genistein (4′,5,7-trihydroxyisoflavone) and daidzein (4′,7-dihydroxyisoflavone), were isolated from *H. erinaceum* mycelium for the first time by HSCCC.

## 2. Results and Discussion

### 2.1. HPLC Analysis

**Figure 1:**
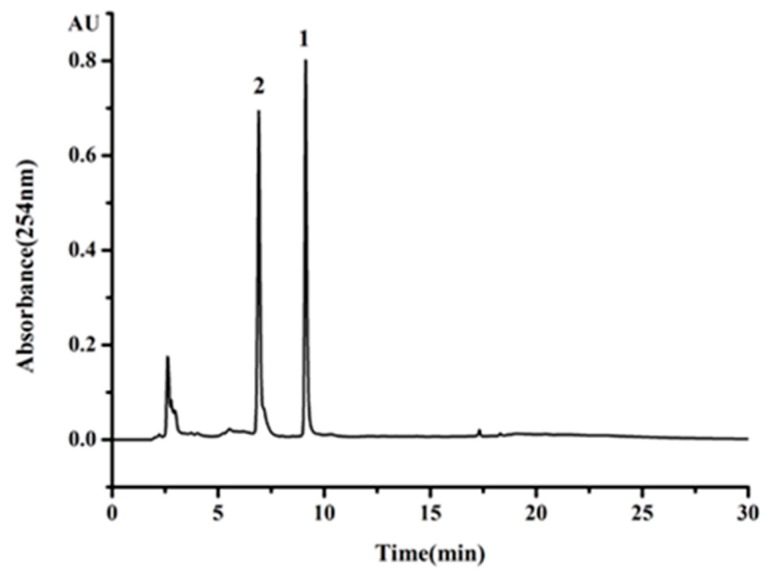
HPLC chromatograms of *H. erinaceum* mycelium crude extract (HEM-E-E). Peak number 1 and 2 refer to compounds **1** and **2**.

Crude extract from *H. erinaceum* mycelium, named as HEM-E-E, was analyzed by HPLC. As showed in Figure 1, compounds **1** and **2** were two main components in HEM-E-E. Compounds **1** and **2** were set as target compounds in further HSCCC separation of HEM-E-E. 

### 2.2. Selection of Two-Phase Solvent System

Choosing a suitable two-phase solvent system is a very important step in HSCCC experiment. The partition coefficient (K) and retention of the stationary phase are key factors for HSCCC separation [20]. The K value usually reflects the distribution between two mutually equilibrated solvent phases. A small K value elutes the solute close to the solvent front with lower resolution. A large K value tends to give better resolution but broader, more dilute peaks [20,27]. The retention of the stationary phase is accomplished by a combination of coiled column configuration and the planetary motion of the column holder. Therefore, the successful separation of HSCCC mainly depends on the selection of the two-phase solvent system. For the target compounds, suitable K values for HSCCC are 0.5 ≤ K ≤ 2.0 and the separation factor (α) between two components should be greater than 1.5 [20,28]. To select a suitable solvent system, several solvent systems were tested in this study. The K and α values in different solvent systems were listed in Table 1. At first, the n-hexane-ethyl acetate/methanol water (HEMWat) system was tested, which can be used for analyses over a wide range of polarity [29,30]. As shown in Table 1, the K values of compounds **1** and **2** in HEMWat (1:1:1:1, 3:2:3:2, 4:5:4:5, *v*/*v*/*v*/*v*) were lower than 0.5, which meant compounds **1** and **2** were mainly distributed in the lower phase. This phenomenon indicated that the system’s polarity was too high, compared to the target compounds. We tried *n*-hexane-methanol-water and ethyl acetate-methanol-water in different composition, but the K values were still lower than 0.5 (data not shown). Then the chloroform/methanol/water (ChMWat) system, which is extremely useful for separations of various natural products with moderate hydrophobicity, was tested [29,30]. When the solvent system was changed to ChMWat (4:4.5:2.5, 5:4:2, 5:5:2, *v*/*v*/*v*), KU/L values of compounds **1** and **2** increased. The results showed that the solvent systems were suitable for the separation of compound **2**. However, the K values for compound **1** were too large, which caused a long time for elution and low resolution. Based on the above data, systems of chloroform-dichloromethane-methanol-water (4:1.5:2:2, 4:2:3:2, 4:4.5:2:5, *v*/*v*/*v*/*v*) were tested. The K values (0.71~0.87 for compound **1** and 0.56~0.91 for compound **2**) were suitable for separation of compounds **1** and **2**. These three solvent systems were selected for further research.

In order to improve the retention of the stable phase, the settling time of the solvent system should be less than 20 s [20]. The settling time of three solvent systems was 24, 14 and 19 s, respectively (Table1). The shorter settling time means a higher retention of the stationary phase. In this study, the solvent system chloroform-dichloromethane-methanol-water (4:2:3:2, *v*/*v*/*v*/*v*) with a settling time of 14 s was selected for further HSCCC separation. Several flavonoids were reported to be separated from the seeds of *Vernonia anthelmintica Willd* by HSCCC using a two-step operation. The two solvent systems were chloroform–dichloromethane–methanol–water (2:2:3:2, *v*/*v*/*v*/*v*) and 1,2 dichloroethane–methanol–acetonitrile–water (4:1.1:0.25:2, *v*/*v*/*v*/*v*) [31], respectively. In this study, we used chloroform-dichloromethane-methanol-water (4:2:3:2, *v*/*v*/*v*/*v*) and separated two isoflavones from *H. erinaceum* mycelium using one-step operation. 

### 2.3. HSCCC Separation

We expect that the suitable two-phase solvent system, chromatographic parameters including flow rate, rotary speed and column temperature may also affect the separation of HSCCC [32]. In this study, the upper and lower phase of chloroform-dichloromethane-methanol-water (4:2:3:2, *v*/*v*/*v*/*v*) system was used as the stationary phase and mobile phase, respectively. The effects of flow rate on separation of the target compounds by HSCCC were shown in Table 2. Both separation time and stationary phase retention decreased with the increase of the mobile phase flow rate. Low flow rate (1 mL/min) could increase to the retention of the stationary phase (67.8%), but it also extended the separation time (320 min). High flow rate (3 mL/min) could decrease the separation time (200 min). However, it decreased the retention of stationary phase (54.3%) and the purity of the target compounds. The retention levels of the stationary phase for a given flow-rate of the mobile phase will greatly contribute to the application of HSCCC. The flow rate is a key parameter that influences the chromatographic behavior after all other conditions are set [33]. Based on the separation time, the stationary phase retention and the purity of the target compounds, 2 mL/min was selected as the optimized flow rate. In this condition, the purity of the target compounds was high, the retention of the stationary phase was 65% and the separation time was 250 min. Our results are consistent with other research that shows that HSCCC is an effective method to isolate isoflavones or flavonoids from raw material [34,35]. 

The rotary speed could also affect the separation time and the stationary phase retention. Low rotary speed reduces the volume of the stationary phase that is retained in the column, which leads to low chromatographic resolution and purity of targeted compounds. However, the high rotary speed might produce excessive sample band broadening due to the violent pulsation in the column [36]. The optimized HSCCC condition for separation of HEM-E-E was 900 rpm (rotary speed) and 20 °C (column temperature).

**Figure 2:**
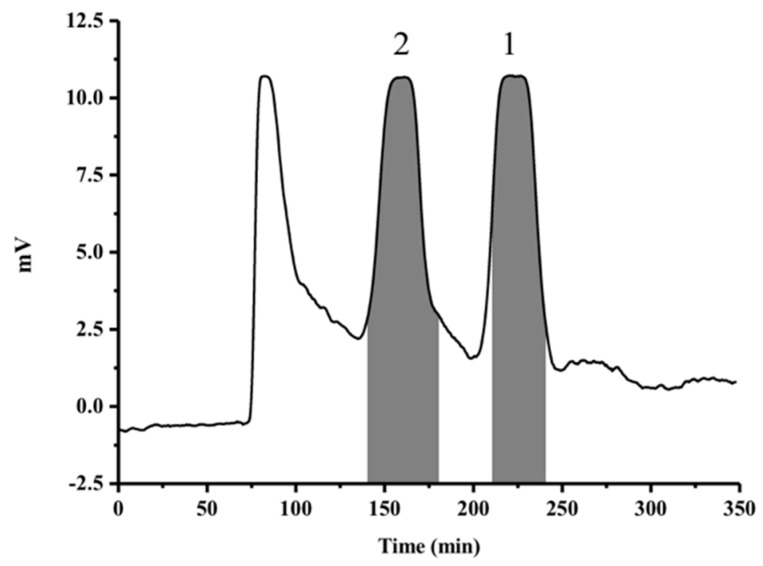
HSCCC chromatogram of HEM-E-E under the optimized condition. The upper phase of chloroform-dichloromethane-methanol-water (4:2:3:2, *v*/*v*/*v*/*v*) system was used as the stationary phase and lower phase of these solvent system was used as mobile phase. HSCCC condition was as follows: flow rate 2.0 mL/min, column temperature 20 °C, sample loading 10 mL, sample content 150 mg/10 mL, detection wavelength = 254 nm, rotary speed= 900 rpm. Peak number 1 and 2 refer to compounds **1** and **2**.

**Figure 3:**
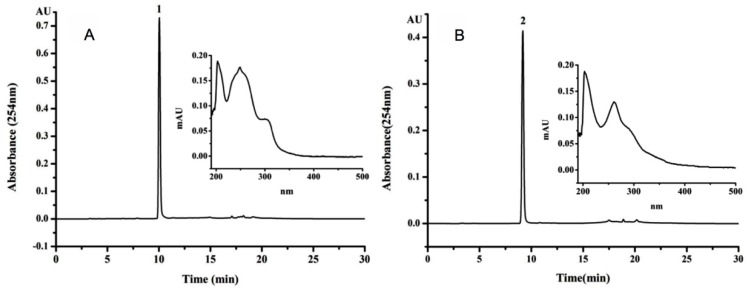
HPLC chromatograms of compound **1** (**A**) and compound **2** (**B**) and UV wavelength scanning of compounds **1** and **2** (inside). Peak number 1 and 2 refer to compounds **1** and **2**.

Under the optimized HSCCC conditions, an appropriate retention percentage of the stationary phase was 64.5%, and the purified target compounds **1** and **2** were obtained (Figure 2). About 23 mg compound **1** and 18 mg compound **2** were yield from 150 mg HEM-E-E. As shown in Figure 3, the purity of compounds **1** and **2** were 95.7% and 97.3%, respectively. It was reported that four isoflavones including daidzein and genistein was separated by HSCCC under a linear gradient elution, using a solvent system composed of n-hexane-ethyl acetate-1-butanol-methanol-water [37]. In another research, daidzein and genistein was separated from *Trifolium pratense L.* by HSCCC using the solvent system of *n*-hexane-ethyl acetate-ethanol-water [38]. In this manuscript, we developed a new solvent system (chloroform-dichloromethane-methanol-water 4:2:3:2 (*v*/*v*/*v*/*v*)) for the separation of genistein and daidzein. It offered higher yields of genistein and daidzein compared to previews research.

### 2.4. Identification of Chemical Structure 

The chemical structures of the compounds **1** and **2** were analyzed by IR, MS, UV and NMR chromatography. 

The structural data of the compound **1** are listed as follows: The ESI-MS showed the pseudo molecular ion [M + H]^+^ peak at *m*/*z* 271.2, corresponding to molecular formula of C_15_H_10_O_5._ The IR absorption bands at 3409.97 cm^−1^ indicated the presence of O-H; the absorption bands at 1615.64, 1650.44 cm^−1^ indicated the presence of C=O; the absorption bands at 1519.11cm^−1^ indicated the presence of aromatic ring; the absorption bands at 1274.23–1043.52 cm^−1^ indicated the presence of C-O. The UV spectrum showed conjugated groups by presenting maximum absorptions at 209 and 254 nm.

The structural data of the compound **2** are listed as follows: The ESI-MS showed the pseudo molecular ion [M + H]^+^ peak at *m*/*z* 255.2, corresponding to molecular formula of C_15_H_10_O_4._ The IR absorption bands at 3219.56 cm^−1^ indicated the presence of O-H; the absorption bands at 1632.21, 1606.62 cm^−1^ indicated the presence of C=O; the absorption bands at 1460.88 cm^−1^ indicated the presence of aromatic ring; the absorption bands at 844.02 cm^−1^ indicated the presence of =C-H on the benzene ring; the absorption bands at 1239.76 and 1193.19 cm^−1^ indicated the presence of C-O. The UV spectrum showed conjugated groups by presenting maximum absorptions at 204, 240 and 299 nm. 

Their spectroscopic data for ^1^H-NMR (400MHz) and ^13^C-NMR (100MHz) were summarized in Table 3.

**Figure 4:**
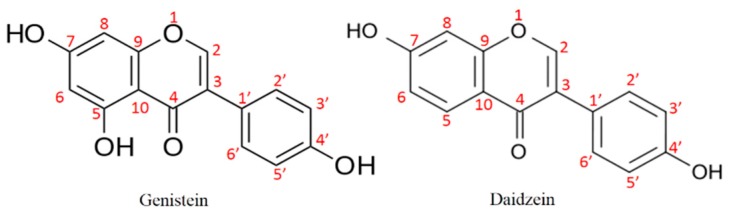
Chemical structures of genistein and daidzein.

The molecular formulas of compounds **1** and **2** were established as C_15_H_10_O_5_ and C_15_H_10_O_4_, respectively. Based on the IR and ^1^H and ^13^C-NMR spectra data, compounds **1** and **2** were identified as genistein (4′,5,7-trihydroxyisoflavone) and daidzein (4′,7-dihydroxyisoflavone), respectively. Our results were consistent with the NMR spectra data of genistein and daidzein in other studies [38,39,40]. Their chemical structures were shown in Figure 4. Genistein and daidzein were two isoflavones isolated from *H. erinaceum* mycelium.

## 3. Experimental Section 

### 3.1. HSCCC Apparatus

HSCCC was performed on a TEB 300A (Tauto Biotechnique Company, Shanghai, China) high-speed counter-current chromatography apparatus. The apparatus consisted of three preparative coils connected in series (the inner diameter of tube, 1.5 mm; total volume, 280 mL) and a 10 mL sample loop. The revolution radius was 5 cm, and the β value was varied from 0.5 at the internal terminal to 0.8 at the external terminal (β = r/R, where r is the distance from the coil to the holder shaft, and R is the distance between the holder axis and the central axis of the centrifuge ) [41]. The rotation speed was ranged from 0 to 1000 rpm. The system was equipped with a model TBP-50A constant-flow pump (Tauto Bioteh, Shanghai, China), a model UV-500 detector (XUYUKJ Instruments, Hangzhou, China) operating at 254 nm, and a model N2000 workstation (Zhejiang University, Hangzhou, China). DC-2010 constant temperature-circulating implement (Hanagzhou Dawei Instrument, Hangzhou, China) was used to adjust the experimental temperature. 

### 3.2. Reagents and Materials

All organic solvents for HSCCC (analytical grade) were purchased from Shanghai Lingfeng Chemical Reagent Co. Ltd. (Shanghai, China). Acetonitrile used for HPLC analysis (chromatographic grade) were purchased from Tianjin Shiled Excellence Technology Co., Ltd. (Tianjin, China). Water was commercial ultrapure water. *H. erinaceus* (Bull.: Fr.) Pers. mycelium powder was purchased from Beijing Fuerkang Biotechnology research institute (Beijing, China) in August 2016. The scientific name was identified by one of the authors (Peilong Sun). The voucher specimen (ZJUT13000) was deposited at the Herbarium College of Pharmacy in Zhejiang University of Technology.

### 3.3. Preparation of H. erinaceum Mycelium Extracts

*H. erinaceum* mycelium powder (1000 g) was extracted three times with 95% ethanol (4L) under reflux for 4 h and at 80 °C using a stir bar. After removing ethanol by vacuum distillation at 55 °C, 76 g obtained ethanol extract was suspended in water (100 mL), and was extracted by petroleum ether and ethyl acetate in sequence. Ethyl acetate fraction was vacuum distilled, and 25 g crude extract was yield. The crude extract was named as HEM-E-E and stored at 4 °C for the HSCCC separation.

### 3.4. Selection of Two-Phase Solvent 

Two-phase solvent systems were selected on the base of the partition coefficient value (K) of the two target compounds. Three solvent systems: (1) *n*-hexane-ethyl acetate-methanol-water systems (*v*/*v*/*v*/*v*, 1:1:1:1 or 3:2:3:2 or 4:5:4:5), (2) chloroform-methanol-water systems (*v*/*v*/*v*/*v*, 4:4.5:2.5 or 5:4:2 or 5:5:2) and (3) chloroform-dichloromethane-methanol-water systems (*v*/*v*/*v*/*v*, 4:1.5:2:2 or 4:2:3:2 or 4:4.5:2:5) were investigated. The K values were determined by HPLC as follows: HEMP-E-E (5 mg) was added to the equilibration of two-phase solvent system, followed by vigorous shaking for 1 min. After two phases were completely separated, 1 mL of each phase was evaporated to dryness, dissolved in 1 mL of acetonitrile and the K values were determined by HPLC analysis. The peak areas of the upper phase and the lower phase were recorded as A_U_ and A_L_, respectively. K value was obtained by the equation: K = A_U_/A_L_. The HPLC conditions were described in Section 3.7. 

### 3.5. Preparation of the Two-Phase Solvent System and Sample Solution

The selected two-phase solvent system composed of chloroform-dichloromethane-methanol-water (*v*/*v*/*v*/*v*, 4:2:3:2) was used for HSCCC separation. The solvent system in a separatory funnel was violently shaken for thorough mixing. After equilibration, the two phases were separated and degassed by sonication for 15 min before used. The lower and upper phase were used as the mobile and stationary phase, respectively. About 150 mg of HEM-E-E were dissolved in the solvent mixture containing 5 mL the lower phase and 5 mL upper phase.

### 3.6. HSCCC Separation

Head-tail elution was performed for the separation of HEMP-E-E. The coiled column was first entirely filled with the upper phase of the solvent system. Then the apparatus was rotated at a speed of 900 rpm, and the lower phase was pumped into the column at a flow rate of 2 mL/min. When the hydrodynamic equilibrium was achieved, as indicated by a clear mobile phase eluting at the tail outlet. About 10 mL of HEMP-E-E solution was injected into the separation column through the injection valve. The separation temperature was controlled at 20 °C. The effluent was continuously monitored at 254 nm, and collected using a fraction collector set at 5 min for each tube. Each fraction was collected according to the chromatogram and evaporated under vacuum. Three HSCCC fractions were obtained, the first fraction contained some impurities (confirmed by HPLC analysis, data not shown), were no longer investigated. The other two fractions are set as two target compounds and named as compounds **1** and **2**, respectively. 

### 3.7. HPLC Analysis of HEM-E-E and Its HSCCC Fractions

The analytical HPLC equipment was a Waters 1525 system consisting of a Waters 1525 Binary pump, a Waters 2487 UV-vis Photodiode array detector, a Waters 2707 injection valve with a 20 μL loop, and a Waters HPLC workstation (Waters, Milford, MA, USA). The column applied in this work was a XTerra MS C18 column (250 mm × 4.6 mm, 5 μm, Waters, USA). The system run with a gradient program at 1 mL/min, and two solvents acetonitrile (A) and water (B) with the following gradient combinations: 0–10 min 30% B; 10–20 min, 30–60% B; 20–25 min, 60–90% B; 25–30 min, 90–60 % B; The eluent was monitored at 254 nm, and the purity was calculated by the target analytic peak area divided by the total peak area (unitary area method).

### 3.8. Identification of HSCCC Peak Fractions

The UV-vis spectra were recorded by a UV-1900 spectrophotometer (Puxi, Beijing, China) using a 1 cm path length cell with absorption wavelength at 254 nm. 

The IR spectrum was recorded in KBr disc and the spectrum was scanned from 400 to 4000 cm^−1^ with a 6700 Nicolet Fourier transform-infrared spectrophotometer (Madison, WI, USA).

ESI-MS was performed by Waters SQD2 mass spectrometer (Waters, Milford, MA, USA), operating in positive mode. The MS conditions were as follows: capillary voltage 3 kV and temperature maintained at 300 °C, cone voltage 40 V, Mass-scan range were measured from *m*/*z* 50 to 1000, source temperature 120 °C, the gas flow rate for cone and desolvation (N_2_) were 500 mL/min. Mass data in this manner provided for the collection of information of intact precursor ions. 

In addition, two purified compounds were concentrated to dryness under reduced pressure and lyophilized, followed by dissolution in deuterated methanol (CH_3_DO) for NMR analysis. The ^1^H and ^13^C spectra were obtained on a Bruker Avance III 400 MHz NMR spectrometer (Bruker Biospin Co., Billerica, MA Rheinstetten, Germany). ^1^H-nuclear magnetic resonance (NMR) and ^13^C-NMR spectra were obtained at the center of analysis, Shanghai Microspectrcum Chemical Technology Service Co. Ltd. (Shanghai, China).

## 4. Conclusions

In this study, the upper and low phase of chloroform-dichloromethane-methanol-water at a volume ratio of 4:2:3:2 (*v*/*v*/*v*/*v*) was selected as the mobile and stationary phase, and the separation condition of *H. erinaceum* mycelium crude extract (named HEM-E-E) were selected as follow: flow rate 2.0 mL/min, rotary speed 900 rpm, column temperature 20 °C. Under the optimized HSCCC conditions, 23 mg compound **1** with the purity of 95.7% and 18 mg compound **2** with the purity of 97.5 % were isolated from 150 mg HEM-E-E. These two compounds were confirmed as genistein (4′,5,7-Trihydroxyisoflavone) and daidzein (4′,7-Dihydroxyisoflavone). To the best of our knowledge, this is the first report in which two isoflavones, genistein and daidzein, are isolated and discovered from *H. erinaceum* mycelium. The results also demonstrated that HSCCC method is a powerful tool for the quick and efficient separation and purification of bioactive compounds from natural products.

## Figures and Tables

**Table 1:** The partition coefficient K, separation factor α and settling time of target components in different solvent systems.

No	Solvent System	Ratio (*v*/*v*)	K Values ^a^		α ^b^	Settling Time
			Compound 1	Compound 2		
1	*n*-hexane-ethyl acetate-methanol-water	1:1:1:1	-	0.24	/	/
2	*n*-hexane-ethyl acetate-methanol-water	3:2:3:2	-	0.27	/	/
3	*n*-hexane-ethyl acetate-methanol-water	4:5:4:5	-	0.25	/	/
4	chloroform-methanol-water	4:4.5:2.5	2.87	0.46	6.23	/
5	chloroform-methanol-water	5:4:2	4.36	1.26	3.46	/
6	chloroform-methanol-water	5:5:2	3.45	0.83	4.15	/
7	chloroform-dichloromethane-methanol-water	4:1.5:2:2	0.84	0.91	1.08	19 s
8	chloroform-dichloromethane-methanol-water	4:2:3:2	0.87	0.56	1.55	14 s
9	chloroform-dichloromethane-methanol-water	4:4.5:2:5	0.71	0.63	1.12	24 s

^a^ K values expressed as: A_U_/A_L_, where A_U_ and A_L_ are the peak of target compound in the upper and lower phase respectively. ^b^ α expressed as: separation factor between two target compounds K_1_/K_2_ or K_2_/K_1_. “-” stand for that the K value was too small.

**Table 2:** Separation time, stationary phase retention and purities of the two target compounds by high-speed counter-current chromatography (HSCCC) as affected by flow rate.

Flow-Rate (mL/min)	Separation-Time (min)	Retention (%)	Purity (%)	
			Compound 1	Compound 2
1	320	67.8	94.2	96.5
2	250	64.5	95.7	97.3
3	200	54.3	92.4	93.5

**Table 3:** ^1^H (400 MHz) and ^13^C-NMR (100 MHz) spectroscopic data of genistein and daidzein. ^a,b^

Pos.	Genistein		Daidzein	
	δ_C_	δ_H_	δ_C_	δ_H_
2	154.77	8.30 (1H, s, H-2)	154.68	8.13 (1H, s, H-2)
3	124.71		125.95	
4	182.23		178.18	
5	163.84		128.52	8.06 (1H, d, *J* = 8.83Hz, H-5)
6	100.10	6.21 (1H, s, H-6)	116.52	6.94 (1H, dd, *J* = 2.24, 8.83Hz, H-6)
7	165.92		164.60	
8	94.27	6.33 (1H, s, H-8)	103.23	6.86 (1H, d, H-8)
9	159.69		159.80	
10	106.28		118.20	
1′	123.29		124.29	
2′	131.38	7.36 (1H, d, *J* = 8.48Hz, H-1′)	131.42	7.37 (1H, d, H-1′)
3′	116.25	6.84 (1H, d, *J* = 8.50Hz, H-2′)	116.22	6.84 (1H, d, H-2′)
4′	158.81		158.69	
5′	116.25	6.84 (1H, d, *J* = 8.50Hz, H-3′)	116.22	6.84 (1H, d, H-3′)
6′	131.38	7.36 (1H, d, *J* = 8.48 Hz, H-4′)	131.42	7.37 (1H, d, H-4′)

^a^ Chemical shifts in ppm, coupling constants in 400 Hz. ^b^ Genistein and Daidzein were measured in CH_3_DO.
